# Design and Implementation of an Electromagnetic–Capacitive Coupling Mechanism-Based Material Young’s Modulus Measurement System

**DOI:** 10.3390/ma19091731

**Published:** 2026-04-24

**Authors:** Zhuo Liu, Xuemei Lu, Heng Li, Baoqing Nie

**Affiliations:** 1 Jiangsu Key Laboratory of Biophotonics, Suzhou City University, Suzhou 215104, China; zhuoliuz@163.com; 2 School of Electronic and Information Engineering, Soochow University, Suzhou 215006, China; hengli0577@163.com; 3 Technology Research Institute, Suzhou LZY Technology Company Ltd., Suzhou 215153, China

**Keywords:** young’s modulus, electrodynamic drive, capacitance detection, measurement system, transient excitation, material mechanical characterization

## Abstract

In mechanical material evaluation and biomechanical studies, Young’s modulus is commonly used to describe the elastic response of materials. Existing measurement approaches are mainly based on contact loading or large-scale experimental instruments, which may limit excitation controllability and system integration in practical applications. In this work, a Young’s modulus measurement system based on electromagnetic excitation and capacitive sensing is designed and experimentally implemented. The system is composed of an electromagnetic driving unit and a capacitive sensing unit. In the driving unit, a coaxial copper wire coil is arranged with a ring-shaped neodymium–iron–boron permanent magnet assembly. When a square-wave electrical signal is applied, the coil generates a Lorentz force, which produces transient mechanical excitation on the tested sample. The resulting micro-scale deformation of the material surface is monitored using a coaxial passive capacitive sensor. The sensor records the relative capacitance variation (ΔC/C_0_) induced by deformation during excitation. Based on the measured capacitance response, a force–capacitance coupling model is established to relate the electrical signal to the mechanical behavior of the material, enabling the inverse calculation of Young’s modulus. Commercial standard hardness blocks were used for system calibration and performance verification. The experimentally obtained Young’s modulus values are consistent with reference data within an acceptable deviation range, indicating that the proposed system can be used for quantitative evaluation of elastic properties. Due to its compact configuration and controllable excitation, the system is suitable for non-invasive surface mechanical characterization of soft materials, including biological tissues.

## 1. Introduction

Accurate characterization of mechanical properties remains a fundamental requirement in materials science and engineering [1,2]. Among various mechanical parameters, Young’s modulus is widely used to describe the elastic response and stiffness of materials, and it plays a critical role in engineering material evaluation as well as biomechanical analysis [3,4,5]. In biomedical applications, the mechanical properties of soft tissues such as blood vessels, nerves, and surrounding connective tissues are closely associated with both physiological function and pathological progression. Abnormal alterations in local tissue stiffness have been reported in multiple disorders, including carpal tunnel syndrome, where changes in mechanical state accompany nerve compression and dysfunction [6]. Consequently, the development of quantitative and reliable methods for measuring Young’s modulus in biomaterials is of considerable research and practical interest.

At present, the Young’s modulus of soft and biological materials is commonly evaluated using techniques such as magnetic resonance elastography, ultrasonic elastography, and direct mechanical loading tests. Although these approaches can provide high measurement accuracy, they typically require bulky instrumentation, complex operational procedures, and high experimental costs, along with stringent requirements on loading conditions and testing environments [7]. Conventional tensile or compression tests, while well-defined in classical engineering mechanics, usually involve relatively large contact forces. This makes them less suitable for small-load or non-invasive measurements and limits their applicability in scenarios involving fragile or bio-related materials.

As an alternative non-destructive approach, the Impulse Excitation Technique (IET) determines the dynamic Young’s modulus by analyzing the vibration response of a material subjected to transient excitation. This method has been extensively studied in engineering material testing, with well-established theoretical models and standardized procedures [8,9]. For example, Massara et al. demonstrated that a low-cost, open-source IET system can effectively classify materials and evaluate mechanical parameters using multi-material datasets. In addition, the ASTM E1876 standard [10] provides systematic guidelines for determining dynamic Young’s modulus based on impact excitation and vibration analysis, confirming the reliability of IET in engineering applications. Nevertheless, traditional IET systems mainly rely on mechanical impact excitation and acoustic or vibration sensors, which restrict their excitation controllability, system integration, and structural compactness [10].

To overcome these limitations, non-contact actuation techniques have attracted increasing attention in precision excitation and dynamic mechanical testing, as they can eliminate frictional effects and structural coupling introduced by mechanical contact. Among them, electromagnetic actuation offers a direct and continuously tunable force output driven by electrical signals, enabling precise control of excitation amplitude and temporal characteristics [11]. Electromagnetic actuators based on lorentz force principles efficiently convert electrical excitation into controllable mechanical motion, providing stable and repeatable transient excitation. Previous studies have shown that such actuation schemes exhibit fast dynamic response while maintaining sufficient force output, meeting the basic requirements for material mechanical excitation and testing [12].

For response detection and signal acquisition, capacitive sensing has been widely employed due to its high sensitivity to minute displacements and deformations. Yoo and Choi systematically reviewed the accuracy and stability of various capacitive sensor readout topologies, highlighting their suitability for high-resolution mechanical measurements [13]. Furthermore, Nam et al. proposed a low-noise Δ–Σ analog front-end based on capacitance-switching techniques, significantly improving the detection accuracy of small capacitance variations and providing a circuit-level foundation for precise mechanical parameter sensing [14].

Despite these advances, existing studies indicate that systems relying on a single excitation or detection mechanism often struggle to simultaneously achieve non-invasiveness, high sensitivity, and compact system integration. By combining electromagnetic actuation with capacitive sensing, a complete “excitation–response–parameter inversion” measurement chain can be established. In such a system, a controlled transient mechanical excitation is applied to the material via electromagnetic actuation, inducing small deformations, while the resulting capacitance variations are continuously monitored and used to inversely determine Young’s modulus through an appropriate mechanical–electrical model. The effectiveness of capacitive detection in capturing subtle mechanical responses and its potential for biomechanical parameter extraction have been demonstrated in previous studies [15]. Moreover, empirical relationships between Young’s modulus and elastomer hardness indices have been systematically explored in the literature, providing essential reference models for experimental calibration of soft materials [16]. The present work builds upon these foundations by integrating electromagnetic actuation with capacitive sensing in a coaxial configuration, thereby offering a novel, non-invasive pathway for direct Young’s modulus inversion under controllable transient loading [17].

Based on the above considerations, this work presents a Young’s modulus measurement system integrating electromagnetic excitation and capacitive sensing. A coaxial structural configuration is adopted to ensure alignment between the coil axis and the ring magnet axis. Periodic axial reciprocating motion of the excitation coil is employed to deliver stable and repeatable transient mechanical stimulation to the tested material. Simultaneously, the capacitive sensing unit detects deformation-induced capacitance variations with high sensitivity, enabling inverse calculation of Young’s modulus based on established electromechanical coupling models [14]. Standard material samples are used to calibrate and evaluate the system performance, thereby validating the feasibility, stability, and applicable measurement range of the proposed method [18].

To highlight the innovation and quantitative advantages of the proposed system, a comparison with representative existing methods is provided in Table 1. The present electromagnetic–capacitive coaxial system achieves a displacement resolution of 2.5 μm while maintaining low cost and high portability, offering clear superiority for non-invasive soft-material and biological-tissue characterization [19,20].

In contrast to conventional techniques that often require invasive loading or large-scale instrumentation. The proposed electromagnetic–capacitive coaxial measurement system is characterized by its nearly non-invasive nature. Both the Lorentz force actuation and the capacitive sensing operate exclusively at the specimen surface, eliminating the need for mechanical contact or bulky imaging equipment. This configuration not only facilitates rapid laboratory characterization of engineering elastomers under controllable transient loading, but also offers a promising platform for non-invasive mechanical assessment of biological soft tissues. Consequently, the present approach provides a convenient and practical tool that bridges fundamental materials research with potential clinical translation, thereby accelerating scientific investigation in biomechanics and soft-matter engineering.

## 2. Materials and Methods

### 2.1. Architecture of a Modular Coaxial Measuring System

**Figure 1:**
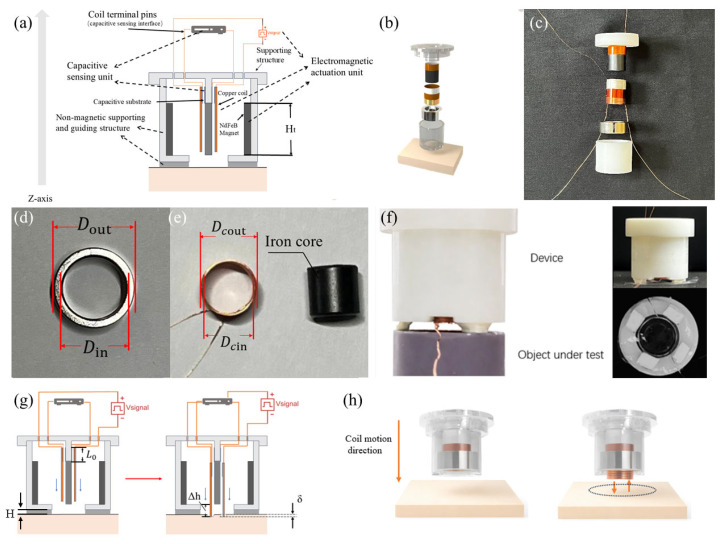
A system that measures the Young’s modulus of tissue. (**a**) The tissue Young’s modulus measurement system’s schematic diagram. 3D-printed housing, a polyimide (PI) sheet with silver paste applied to both surfaces, an iron core, a copper coil, an annular permanent magnet, and copper lead wires for the pole plates are the functional layers arranged from top to bottom. (**b**) The system’s exploded 3D perspective. This demonstrates the spatial layout between the coil and magnet assemblies. (**c**) The system’s actual assembly diagram, which shows how important parts from the exploded view and the supporting structures above and below are actually aligned. (**d**,**e**) Real photos of the iron core, magnet, and coil with the magnet’s and coil’s measurements marked. (**f**) Close-up photos of the system; the top and front views are shown in the two pictures on the right. (**g**) Schematic representation of the driving unit in the motion process in the experimental system. (**h**) A 3D-modeled image that makes the mobility status of the system very evident.

The material hardness assessment method suggested in this study uses a modular, coaxially symmetrical structural architecture, as seen in Figure 1a. An electromagnetic driving unit, a capacitive sensor unit, and a non-magnetic support and guidance framework make up the entire system. The measurement core of the system is axial transient excitation. Under the influence of a magnetic field, the coil produces Lorentz forces when it is powered, allowing reciprocating motion in a predetermined z-axis direction to provide axial mechanical excitation to the tested material [21]. In terms of structure, non-magnetic 3D-printed brackets at both ends function as load-bearing and positioning structures that define coil movement routes and secure magnet placement. This design guarantees spatial stability and axial alignment of functional components while preventing ferromagnetic interference with magnetic field distribution [22,23].

The transparent upper and lower structures that correspond to the previously indicated support brackets are visible in the exploded view in Figure 1b. These brackets, which are made from non-magnetic resin using 3D printing, have internal dimensions that are exactly suited to the magnet’s profile, guaranteeing coaxial alignment and stability during assembly. The structural basis for producing a steady, controllable magnetic field is provided by the electromagnetic drive unit, which consists of a neodymium iron boron (N38) ring-shaped permanent magnet coaxially arranged with a copper wire coil along the axial direction. Building on this, the magnetic ring and its supporting structure depicted in Figure 1b are physically demonstrated in Figure 1c. This shows the actual assembly state and offers visual confirmation of the structural design’s viability. Figure 1d shows the geometric dimensions of the magnetic ring: outer diameter $D_{out}$ = 16 mm, inner diameter $D_{in}$ = 12 mm, and thickness $H_{t}$ = 6 mm. The magnet’s axis is precisely aligned with the device’s overall z-axis, and it is firmly anchored inside a 3D-printed resin support. Within the magnetic ring’s inner bore, this configuration produces a magnetic field distribution that is roughly axisymmetric. The coil assembly is located inside the magnetic ring’s inner bore in Figure 1e. It is wound with enameled copper wire of diameter $H_{c}=0.35\;\mathrm{mm}$, comprising 78 turns with a total length of approximately 2.72 m, forming an annular coil with axial height $H_{cz}=9\;\mathrm{mm}$. The inner and outer diameters of the coil are $D_{cin}=10\;\mathrm{mm}$ and $D_{cout}=10.90\;\mathrm{mm}$, respectively. Hence, the radial gap between the coil’s outer edge and the inner bore of the magnetic ring is $L_{\mathrm{gap}}=\frac{D_{\mathrm{in}}-D_{\mathrm{cout}}}{2}=0.55\;\mathrm{mm}$. This gap provides the required mechanical clearance for the coil’s axial movement while guaranteeing magnetic flux coupling. Furthermore, the black cylindrical core at the axis center optimizes the magnetic circuit structure and improves magnetic coupling, as seen in Figure 1e. This design enhances the magnetic flux density in the coil region, hence amplifying the Lorentz force created by the coil within the annular magnetic field. The measured front view of the built device, as well as its front view and measurement plane view during typical operation, is shown in Figure 1f. From the viewpoints of the main contact interface and the overall structure, this picture offers a clear depiction of the device’s actual operating condition. Notably, the axial height of the coil exceeds the thickness of the magnetic ring by Δh=3.0 mm. The coil extends about 1.5 mm past both ends of the magnetic ring, as shown in Figure 1g. This structural characteristic guarantees that the coil conductor stays inside the magnetic field gradient zone throughout the axial displacement range by ensuring that the coil maintains full overlap with the effective magnetic field region of the magnetic ring during its entire motion. A current-carrying conductor in a magnetic field experiences a force proportional to the current direction and magnetic flux distribution, according to the Lorentz force law. The physical basis for creating a stable, controllable axial electromagnetic drive is provided by this construction.

Schematic illustrations of the device operating during measurement and the corresponding three-dimensional modeling results are presented in Figure 1g and Figure 1h, respectively. The axial displacement behavior along the Z-axis during loading is clearly demonstrated by visualizing the motion of the coil–magnet assembly. This motion pattern confirms that the proposed device achieves well-controlled, single-degree-of-freedom axial motion during measurement, which is consistent with the intended structural design. Such a motion characteristic provides a reliable mechanical foundation for subsequent Young’s modulus inversion based on displacement–force relationships.

After assembly, the coil axis is precisely aligned with the magnetic ring axis and, together with the upper and lower capacitive plate structures, forms an integrated measurement module supported by the non-magnetic framework. This coaxial configuration effectively suppresses eccentric torque and off-axis motion during excitation and measurement, ensuring stable axial actuation and repeatable displacement responses [24]. The coil accomplishes smooth reciprocating motion along the z-axis under non-contact conditions by combining a guiding structure with precisely controlled radial clearance. Structurally, this design optimizes magnetic flux utilization efficiency by avoiding magnetic circuit gaps and maintaining axial symmetry, concentrating the electromagnetic driving force primarily on the axial degree of freedom. This drive mechanism minimizes the contact uncertainties typical in classic mechanical impact structures, permitting the recording of dynamic response data with great reproducibility [9,10,12]. In precision actuators and micro-force excitation systems, similar electromagnetic drive architectures have shown exceptional controllability, dynamic response performance, and high-precision stability. These benefits offer a solid engineering foundation for using this system in situations involving material mechanical excitation [25].

The modular coaxial measurement system was designed, fabricated, and assembled in the Sensor Laboratory (Room 326, Electronic Information Building, Suzhou University, Suzhou, Jiangsu Province, China) under standard laboratory conditions (23 ± 2 °C, 50 ± 10% relative humidity) on a non-magnetic platform to eliminate external magnetic interference.

The system consists of a N38 ring neodymium permanent magnet (AIM Magnet Co., Ltd., Shenzhen, Guangdong Province, China), a driving coil wound with enameled copper wire (standard university-procured laboratory consumable, Suzhou University central procurement), and a central iron core (Ningbo Vastsky Magnet Co., Ltd., Ningbo, Zhejiang Province, China). The coil is driven by a function/arbitrary waveform generator (Siglent Technologies Co., Ltd., Shenzhen, Guangdong Province, China). The capacitive sensing unit is fabricated on polyimide (PI) film substrate (Suzhou Yiteng Electronic Materials Co., Ltd., Suzhou, Jiangsu Province, China) coated with conductive silver paste (SINWE 3706 model, Shenzhen SINWE Technology Co., Ltd., Shenzhen, Guangdong Province, China). The non-magnetic support structures are 3D-printed using Vnici T-T86 resin (Changzhou Vnici Digital Technology Co., Ltd., Changzhou, Jiangsu Province, China) on a REMP3D M2 printer (Nanjing Ruipu Information Technology Co., Ltd., Nanjing, Jiangsu Province, China). Capacitance changes are measured with a Wayne Kerr 6500B precision impedance analyzer (Wayne Kerr Electronics Ltd., Chichester, West Sussex, UK). Calibration was performed using a Mark-10 M5-2 force gauge (Mark-10 Corporation, Copiague, NY, USA) and a standard vernier caliper (Mitutoyo Corporation, Kawasaki, Japan). Commercial Shore Hardness A test blocks (10A–70A) were procured from TMTeck Instrument Co., Ltd. (Beijing, China), and insulating double-sided tape was obtained from 3M China Co., Ltd. (Shanghai, China).

This detailed sourcing ensures full reproducibility of the experimental setup.

### 2.2. Electromagnetic Excitation and Axial Force Derivation and Calculation

**Figure 2:**
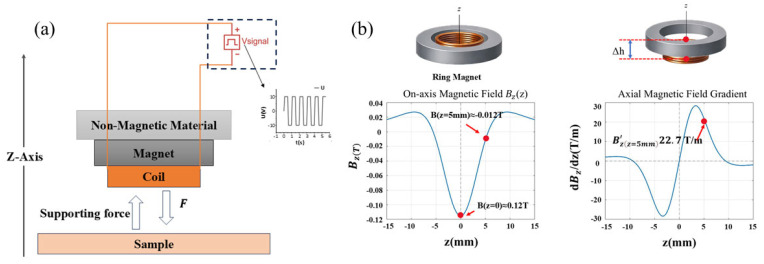
Axial magnetic field and magnetic field gradient modeling and system operation. (**a**) Schematic diagram of forces acting on the system and the sample under the excitation signal. (**b**) Graph of axial magnetic field $B_{z}$, magnetic field gradient curve, and magnetic field gradient annotation at the offset position $z_{0}$ ≈ 5 mm.

A schematic diagram of the mechanical interaction between the apparatus and the test specimen when an excitation load is applied is shown in Figure 2a. The relationship between the electromagnetic drive unit, the measuring apparatus, and the test specimen is clearly shown in this diagram. The device uses a configuration that combines an energized coil with a ring-shaped neodymium–iron–boron permanent magnet to produce variable axial loading [26]. The axial magnetization of the disk provides an analytical expression for the axial magnetic flux density at any point along the permanent magnet’s z-axis [27,28]:

(1) $$B_{\mathrm{disk}}\left(z\right)=\frac{B_{r}}{2}\left(\frac{z+t/2}{\sqrt{{\left(z+t/2\right)}^{2}+R^{2}}}-\frac{z-t/2}{\sqrt{{\left(z-t/2\right)}^{2}+R^{2}}}\right)$$

In Equation (1), $B_{\mathrm{disk}}$ represents the magnetic field strength at the z-axis of the cylindrical permanent magnet, $B_{r}$ denotes the remanence of the permanent magnet (N38 neodymium iron boron magnet), *t* is the axial thickness of the magnet, *R* is the magnet radius, and *z* is the axial distance from the observation point to the geometric center of the magnet. For annular magnets with an inner bore, the magnetic field strength along the z-axis is the difference between the outer and inner rings:

(2) $$B\left(z\right)=B_{\mathrm{disk}}\left(z;R_{o}\right)-B_{\mathrm{disk}}\left(z;R_{i}\right)$$

Here, $R_{i}$ denotes the inner radius of the magnet, and $R_{o}$ denotes the outer radius. Based on the idea of magnetic field superposition, Equation (2) calculates the axial magnetic field distribution of the annular permanent magnet by equating it to the difference between a cylindrical magnet with an outer radius and one with an inner radius. The magnetic field and gradient calculations in this study are theoretically based on Equation (2), which is a frequently used analytical solution for axisymmetric permanent magnets.

In a finite-length coil, the magnetic field distribution along the axis exhibits significant spatial dependence [29,30]. The coil’s core area has a rather uniform magnetic field that is getting close to its maximum strength. The increased contribution of end-element currents in the finite-length coil keeps the axial magnetic field intensity high toward the coil end faces. Nevertheless, the magnetic field contribution quickly falls, and the overall field intensity drops drastically with distance when the measurement point goes past the end face into the coil’s exterior. The edge effect at the ends of finite-length coils is reflected in this pattern. It has been confirmed in several practical and theoretical investigations and can be quantitatively defined using Biot-Savart Law or analytical formulas for finite-length solenoids [31,32]. After substituting the geometric dimensions and material remanence $B_{r}$ of the magnet, the spatial distribution of magnetic flux density along the axis can be calculated using the analytical magnetic field model for axially uniformly magnetized cylindrical ring permanent magnets. The calculation results indicate that at the geometric center of the magnet, Z=0, the axial magnetic flux density is $B\left(z=0\right)\approx 0.12T$. This result indicates that a finite internal magnetic field still persists in the magnet’s axial region even though it is much smaller than the magnet’s remanence. The magnetic field distribution fulfills $B\left(z\right)=B\left(-z\right)$ because of the magnet’s strong axial symmetry about its geometric center. Thus, at z=0, the first spatial derivative is zero, i.e., ${\left.\frac{\partial B}{\partial z}\right|}_{z=0}=0$, indicating no axial gradient at the center position. The magnetic field strength quickly decreases with axial distance when looking at locations that are distant from the center, like Z=+5mm. $B\left(z=+5\mathrm{mm}\right)\approx -0.012T$, with an amplitude of roughly 0.012 T, is the result of calculations (the sign is only decided by the choice of positive coordinate direction). At this location, there is also a noticeable spatial gradient along the axial direction. We differentiate the analytical expression for the axial magnetic field with respect to z and use the same geometric and material parameters to derive the spatial distribution features of the axial magnetic field gradient. We differentiate with respect to z using Equation (1) and compute:

(3) $$\frac{\partial B}{\partial z}=\frac{B_{r}}{2}\left(\frac{d}{dz}f_{+}\left(z\right)-\frac{d}{dz}f_{-}\left(z\right)\right)$$

among which

(4) $$f_{\pm }\left(z\right)=\frac{z\pm \frac{L}{2}}{\sqrt{{\left(z\pm \frac{L}{2}\right)}^{2}+R^{2}}}$$

A closed-form solution for the axial magnetic field gradient can be obtained:

(5) $$\frac{\partial B\left(z\right)}{\partial z}=\frac{B_{r}}{2}\left[\frac{R^{2}}{{\left[{\left(z+\frac{L}{2}\right)}^{2}+R^{2}\right]}^{3/2}}-\frac{R^{2}}{{\left[{\left(z-\frac{L}{2}\right)}^{2}+R^{2}\right]}^{3/2}}\right]$$

The contributions of the magnet’s upper and lower magnetized end faces to the axial magnetic field gradient are represented by the two terms in Equation (5). The main spatial influence of the two terms alternates continually as the observation point shifts along the axial direction due to variations in its relative distance to the two end faces. The magnetic charge at the lower end face dominates the gradient at the lower end face region, whereas the magnetic charge at the higher end face dominates the gradient near the top end face. The contributions from both end faces cancel each other out close to the magnet’s geometric center. As a result, non-monotonic fluctuation and spatial sign reversal are unavoidable in the axial magnetic field gradient. This phenomenon is not caused by modeling anomalies or numerical errors, but rather by the inherent physical process of end-face effects in finite-sized magnets. In this work, the analytical formulas based on Equations (2) and (5) are numerically implemented in the MATLAB (2018b) environment to derive the spatial distribution curve of the axial magnetic field gradient. The axial magnetic field gradient function is constructed in MATLAB, and point-by-point sampling calculations are performed along the axial coordinate to derive the continuous distribution curve of $\frac{\partial B}{\partial z}$ as a function of z (Figure 2b). The contributions from both end faces cancel each other out close to the magnet’s geometric center. As a result, non-monotonic fluctuation and spatial sign reversal are unavoidable in the axial magnetic field gradient. This phenomenon is not caused by modeling anomalies or numerical errors, but rather by the inherent physical process of end-face effects in finite-sized magnets. In this work, the analytical formulas based on Equations (2) and (5) are numerically implemented in the MATLAB environment to derive the spatial distribution curve of the axial magnetic field gradient. This approach avoids discrete mistakes and numerical oscillations caused by finite element meshing by relying solely on analytical formulas. It directly displays the intrinsic analytical properties of the axial magnetic field gradient in finite-length permanent magnets. It is challenging to create a static axial electromagnetic driving force at the magnet center because, as the image illustrates, axial symmetry causes the axial gradient to disappear even though the magnetic field intensity there is non-zero. On the other hand, the magnetic field gradient greatly increases when the coil moves axially away from the symmetry center and into the non-zero gradient region. This gives the coil and the non-uniform magnetic field the coupling conditions they need to produce an effective axial driving force.

When the coil is energized, the axial magnetic dipole moment m of the coil is

(6) $$m=N\times I\times A_{c},\left(A_{c}=\pi {\times r}_{\mathrm{mean}}^{2}\right)$$

where *N* is the number of turns, I is the coil current amplitude, $A_{c}$ is the average cross-sectional area of the coil, and $r_{mean}$ is the average radius of the coil. When the characteristic dimension$\;l_{c}$ of the coil is much smaller than the spatial variation characteristic length $L_{B}$ of the magnetic field, defined as $L_{B}=\frac{\left|B\right|}{\left|\frac{\partial B}{\partial z}\right|}$, such that $l_{c}\ll L_{B}$, the magnetic field can be approximated as having a uniform gradient across the entire effective area of the coil [33,34]. As a result, the force applied to a magnetic dipole in an axial magnetic field gradient can be used to approximate the electromagnetic force acting on the coil. The axial force equation for this force is provided by

(7) $$F_{z}\approx m\times \frac{\partial B}{\partial z}=N\times I{\times A}_{c}\times \frac{\partial B}{\partial z}$$

Significant variations happen in areas with non-uniform magnetic field intensity or when the coil and magnet have comparable diameters [35,36].

In this experiment, the electrical signal driving the coil is a symmetrical square wave. For a symmetrical square wave, the current amplitude (I) in the drive coil equals the RMS current $\left(I_{\mathrm{RMS}}\right)$, i.e., $I=I_{\mathrm{RMS}}=\frac{V_{\mathrm{RMS}}}{R}$ (where $V_{\mathrm{RMS}}$ is the RMS value of the drive voltage). Therefore, when computing the magnetic dipole moment and axial force under square wave drive, putting the peak current value into Equation (6) produces the peak magnetic dipole moment. This experiment takes into consideration the resistance of the coil and signal generator, limiting the actual current in order to precisely determine the peak axial excitation force applied to the sample by the device. The coil resistance is $R_{coil}$ = 2.15 Ω, and the output impedance of the signal generator (Digi-Key SDG1050) is $R_{gen}$ = 50 Ω. Under series connection conditions, the total impedance experienced by the coil is: R = $R_{coil}$ + $R_{gen}$ = 52.15 Ω. Given $V_{RMS}$ = 10 V, the peak current I driving the coil is

(8) $$I=\frac{V_{\mathrm{RMS}}}{R}\approx 0.19\;A$$

The coil has N = 78 turns, with an average radius $r_{mean}$ = 5.55 mm = 5 × 10^3^ m. Therefore,

(9) $$A_{c}=\pi \times r_{\mathrm{mean}}^{2}\approx 9.68\times 10^{-5}\;m^{2}$$

Substitute into Equation (6) to calculate the magnetic moment:

(10) $$m=N\times I\times A_{c}\approx 0.0015\;A\cdot m^{2}$$

Under a non-uniform axial magnetic field gradient, the maximum axial electromagnetic force produced by this magnetic dipole moment is

(11) $$\;F_{z}\approx m\frac{\partial B}{\partial z}\approx 0.034\;N$$

By further comparing the peak coil current with the rated output current of the signal generator under a 50 Ω load, it can be seen that:

(12) $$I=0.19\;A\;<I_{\mathrm{peak}}=\frac{V_{\max}}{R}=0.2\;A$$

Because the coil current did not surpass the signal generator’s rated output during the experiment, the experiment’s viability and the accuracy of the data were guaranteed.

From this, the total excitation applied to the sample can be calculated. Given the sample mass $\left(m_{\mathrm{sample}}=0.00086\;\mathrm{kg}\right)$ and gravitational acceleration $\left(g=9.81\;m/s^{2}\right)$, the gravitational force acting on the sample is

(13) $$G=m_{\mathrm{sample}}\times g\approx 0.0084\;N$$

Therefore, the total effective loading force used for inversion is taken as

(14) $$F=F_{z}+G\approx 0.042\;N$$

### 2.3. Young’s Modulus Inversion Method

This measurement employs a coaxial cylindrical capacitive displacement sensor. Under ideal coaxial approximation, the capacitance at an overlap length L is [37]:

(15) $$C\left(L\right)=\frac{2\pi \varepsilon_{d}L}{\ln\left(b/a\right)}=K\times L\;$$

Here, a and b denote the radii of the inner and outer electrodes, respectively, and $\varepsilon_{d}$ is the dielectric constant of the dielectric layer. The constant K is defined as $K=\frac{2\pi \varepsilon_{d}}{\ln\left(b/a\right)}$. This formula represents the classical expression for a cylindrical coaxial capacitor.

Let the initial overlap length be *L*_0_, and the initial capacitance be $C_{0}=K\times L_{0}$. When displacement causes a capacitance change Δ*C*, the total displacement can be calculated in reverse via a linear relationship:

(16) $$\Delta h=L_{0}\frac{\Delta C}{C_{0}}$$

Only the fraction of displacement that exceeds the spacer height *H* compresses the sample if the equipment has a set spacer height H. Therefore, the effective sample compression is

(17) $$\delta =\Delta h-H=L_{0}\frac{\Delta C}{C_{0}}-H$$

and requires $L_{0}\frac{\Delta C}{C_{0}}>H$ to ensure positive compression.

It should be noted that the linear relationship is established by the edge effects and calibration discussed above, assuming that nearby conductive components, edge electric fields, dielectric inhomogeneities, and parasitic capacitance are disregarded. Calibration should be carried out in real trials to confirm the linear range and coefficient *K* [38]. Under axial loading and assuming uniform compression of the specimen, Young’s modulus is defined as

(18) $$E=\frac{F/A_{s}}{\delta /L_{s}}=\frac{F\;L_{s}}{A_{s}\;\delta }$$

where $A_{s}$ is the contact area between the sample and the coil, $L_{s}$ is the nominal length of the sample, and δ is the axial compression of the sample. Combining δ with the capacitance yields the inversion expression:

(19) $$E=\frac{FL_{s}}{A_{s}\left(L_{0}\frac{\Delta C}{C_{0}}-H\right)}$$

The quantitative relationship used in this experiment is represented by this equation, which states clearly that the quantities *F*, *Ls*, *As*, *L*_0_, *C*_0_, Δ*C*, and *H* must be precisely measured in order to produce *E*. Among them, Young’s modulus *E* is correlated with the relative change in device output capacitance, $\frac{\Delta C}{C_{0}}$, while parameters *F*, *Ls*, *As*, *L*_0_, and *H* are constants.

The resolution of the capacitive sensor is determined by the basic measurement accuracy of the Wayne Kerr 6500B precision impedance analyzer (±0.05% of reading under SLOW speed, 1 V drive level, fully trimmed conditions, and 23 ± 5 °C) [39]. This relative accuracy corresponds to a minimum resolvable relative capacitance change of $\frac{\Delta C}{C_{0}}$ ≈ 5 × 10^−4^ (dimensionless). According to the coaxial capacitor model (Equation (15)), the relative capacitance change is linearly related to the total axial displacement by Equation (16): Δh = $\frac{\Delta C}{C_{0}}L_{0}$. In the present study, the initial electrode overlap length is L_0_ = 5 mm; therefore, the minimum detectable total axial displacement is

(20) $$\delta_{\min}\approx 5\times 10^{-4}\times 5\;\mathrm{mm}=2.5\;\mu m.\;\;\;\;\;$$

After subtracting the fixed preload height H (Equation (17)), the minimum effective sample compression $\delta_{min}$ is likewise approximately 2.5 μm. When the induced effective compression δ under the applied load significantly exceeds this resolution limit (Shore 10A–60A), the inversion model (Equation (21)) yields physically reasonable Young’s modulus values. In contrast, when δ approaches or falls below 2.5 μm (Shore 70A), the uncertainty in $\frac{\Delta C}{C_{0}}$ exceeds the signal amplitude, resulting in deviation from the linear regime or even non-physical negative values, thereby defining the upper limit of the effective measurement range of the present system.

### 2.4. Measurement Platform Calibration and Mechanical Response Verification

**Figure 3:**
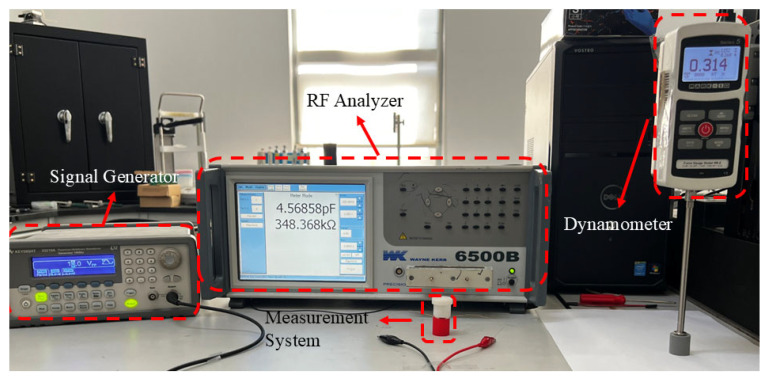
Experimental Test Schematic Diagram.

The experimental platform is calibrated, and the suitability of a commercially available shore A hardness block is confirmed. The total test design is depicted in Figure 3. The experimental measuring platform was built on commercially available Shore hardness standard blocks. To guarantee comparability between samples of different hardness, all reference blocks were evaluated during calibration under the same contact conditions and loading settings. A millimeter-grade force gauge was used to measure each standard block’s force-displacement response during loading. Their corresponding Young’s moduli were obtained by combining this information with geometric parameter conversions.

**Figure 4:**
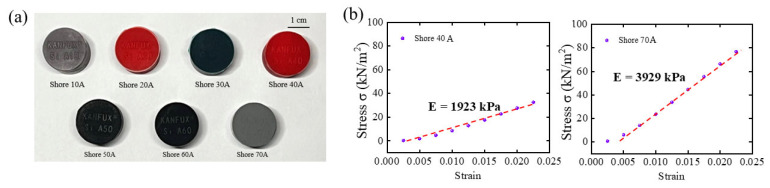
An image of two common hardness elastomers’ stress–strain response curves and commercially available Shore A hardness blocks. (**a**) Seven Shore hardness blocks with steadily rising hardness levels; (**b**) Shore-40A and Shore-70A calibration data curves.

As seen in Figure 4a, this study used seven commercially available Shore A elastomer hardness blocks with nominal hardness values ranging from 10A to 70A as reference samples to calibrate the measurement reliability and applicability of the built experimental apparatus. The stress–strain response curves of two sample elastomers, Shore 40A and Shore 70A, are shown as illustrative examples in Figure 4b. Both materials show stable and roughly linear stress–strain relationships within the minor strain range.

**Figure 5:**
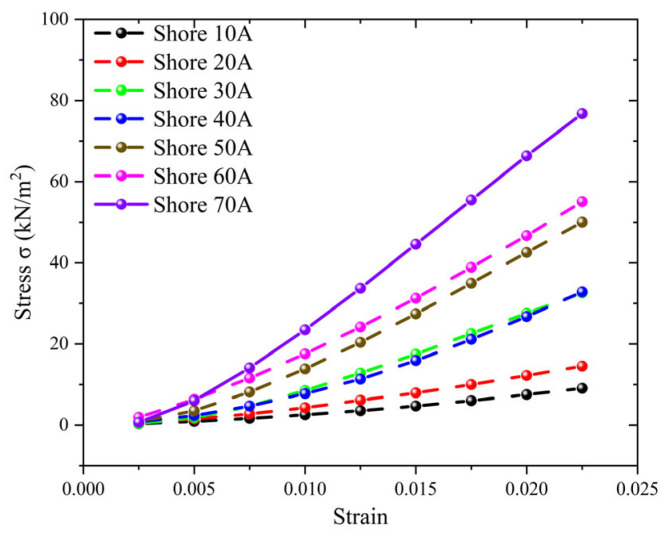
Stress–Strain Curves for Seven Shore Hardness Blocks.

The stress–strain curve’s slope noticeably rises with Shore A hardness (Figure 5), indicating a notable increase in the material’s equivalent stiffness. This finding shows that the experimental platform can consistently produce linear mechanical responses from elastomers with varied Young’s moduli under the imposed stress and measurement conditions. The remaining Shore A hardness samples’ stress–strain curves are not displayed separately because they have the same linear properties. The stress–strain curve’s slope noticeably rises with increasing Shore A hardness, indicating a linear rise in the material’s Young’s modulus. For each hardness sample, a stable linear response region is clearly discernible within the low strain range, where linear fitting of experimental data points demonstrates excellent correlation. The calibration results show that commercially available Shore A hardness blocks, whose stiffness distribution spans the testing platform’s operational range, offer steady, reproducible mechanical responses under the experimental conditions. This demonstrates that the hardness blocks can produce mechanical responses typical of elastomers with different Young’s moduli within the investigated strain range, meeting the linearity assumption necessary for Young’s modulus extraction.

### 2.5. Preparation Process of the Capacitive Sensing Unit

**Figure 6:**
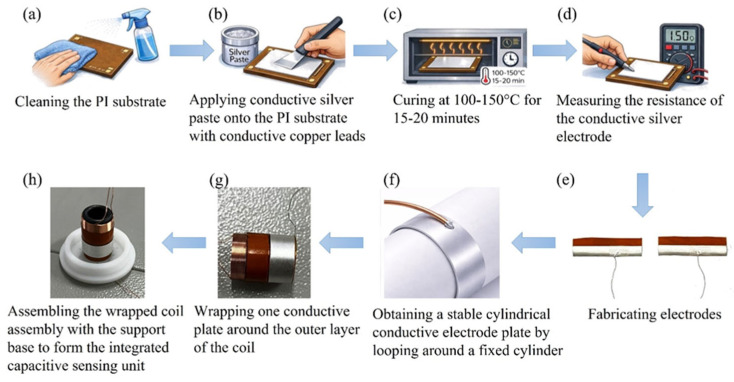
(**a**) Cleaning the PI substrate; (**b**) Applying conductive silver paste onto the PI substrate with conductive copper leads; (**c**) Curing at 100–150 °C for 15–20 min; (**d**) Measuring the resistance of the conductive silver electrode; (**e**) Fabricating electrodes; (**f**) Obtaining a stable cylindrical conductive electrode plate by looping around a fixed cylinder; (**g**) Wrapping one conductive plate around the outer layer of the coil; (**h**) Assembling the wrapped coil assembly with the support base to form the integrated capacitive sensing unit.

Capacitive sensing unit assembly and conductive silver paste preparation. Stable interface bonding between low-resistance conductive pathways and dielectric substrates is essential for creating high-performance capacitive substrates. The conductive layer material for capacitive electrodes in this work is conductive silver paste based on silver nanoparticles. This silver paste’s superior rheological characteristics and process adaptability allow for consistent brush coating deposition onto polymer surfaces. It creates a continuous, dense conductive network after low-temperature curing, guaranteeing steady electrical performance [40,41,42,43]. In order to balance electrode layer adhesion and conductivity while avoiding substrate thermal deformation, conductive silver paste was applied to the PI substrate surface under regulated conditions and then thermally cured at 100–150 °C. Two conductive substrates were looped around a fixed cylinder to provide a stable cylindrical arrangement following electrode manufacturing. After curing, one substrate was further wrapped around the outer layer of the coil, while the other was fastened to a substrate of a non-magnetic support structure. A fully integrated capacitive sensing unit was subsequently created by integrating the wrapped coil assembly with the support base, resulting in synergistic optimization between the electrode layer and structural system (Figure 6). When creating intricate geometries, 3D-printed non-magnetic support structures are essential because they offer structural reinforcement without sacrificing electromagnetic performance [44,45,46].

This study combines conductive silver paste with additive manufacturing technology and employs an optimized support design, providing a scientific rationale and a scalable pathway for the feasible production of high-performance capacitor substrates.

## 3. Results and Discussion

### 3.1. Young’s Modulus Extraction and Experimental Platform Calibration

The self-built testing platform was used to validate the experimental results. Each Shore A hardness sample’s linear intervals were evenly processed using the previously indicated stress–strain data, and linear regression was used to extract the corresponding equivalent Young’s moduli. The Young’s modulus values obtained for different Shore A hardness samples under the same calibration procedure are reported in Table 2. The equivalent Young’s modulus shows a trend of monotonic increase with Shore A hardness. This outcome is consistent with the typical mechanical behavior of elastomeric materials, suggesting that the experimental platform has reliable and consistent calibration capabilities throughout a wide range of stiffness. Note that the initial linear section of the stress–strain curve was the only basis used to extract Young’s modulus. This region was created using a linear fitting quality criterion to enable consistent physical interpretation when comparing samples of varying Shore A hardness ratings. The calibration results confirm that commercially available Shore A hardness blocks can be used in the experimental setup. In order to characterize their corresponding Young’s moduli, the mechanical responses of the commercial Shore A blocks were independently assessed using force-displacement measurements in these calibration experiments [47].

The measured values are in good agreement with the Gent reference values in the low-hardness range (10A–20A), with relative deviations below 15%, indicating that the proposed system has good accuracy for soft materials. In the medium-to-high hardness range (40A–70A), the measured values are systematically lower than the reference values (relative deviations of 10–30%). This discrepancy is consistent with the material characteristics of commercial Shore hardness blocks (mostly silicone rubber or polyurethane, which differ in composition from the ideal natural rubber on which the Gent formula is based). The above results validate the reliability and applicability of the developed measurement system, while also showing that the actual mechanical response of commercial hardness blocks is slightly softer than the theoretical model. This provides a more realistic calibration benchmark for subsequent applications in simulating biological soft tissues. The main sources of the discrepancy include material formulation, filler type, and processing conditions, which are consistent with reports on similar elastomers in the literature (Meththananda et al., 2009) [16].

### 3.2. Applicability Analysis and Calibration Outcomes of the Self-Built System

**Figure 7:**
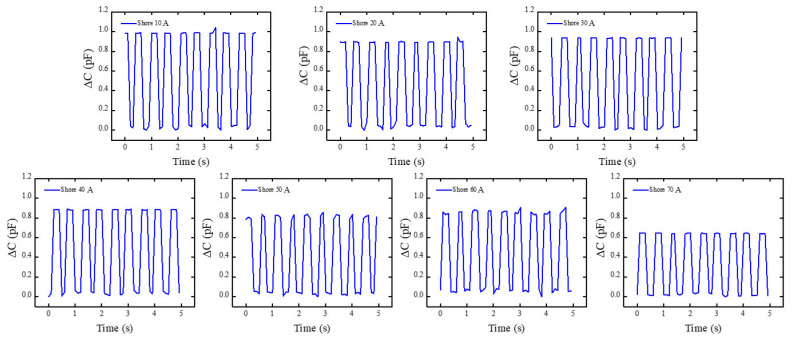
The capacitance’s raw response curve as it changes over time with periodic excitation. It can be noted that the capacitance signal demonstrates remarkable repeatability and stability during each motion cycle, with its variation trend being consistent with the mechanical motion process. This indicates that capacitance variations during periodic mechanical oscillatory motion can be accurately detected by the built-in measurement equipment.

Building on Section 3.1, Shore A hardness block of the same kind was subjected to preset excitation using the self-built experimental apparatus. The platform’s mechanical-electrical coupling capability was verified by monitoring its electrical response. This aims to evaluate the system’s ability to perceive and distinguish differences in the material Young’s modulus. Within this experimental platform, a square wave excitation signal was applied to the coil: a square wave excitation signal with a frequency of 1 Hz and an effective value ($V_{\mathrm{rms}})$. The RF analyzer’s raw electrical response output was concurrently recorded during this procedure. The system’s typical raw signals for several Shore A hardness standard blocks under the same excitation settings are displayed in Figure 7. The system’s typical raw capacitance signals for several Shore A hardness standard blocks (30A, 40A, 50A, and 60A) under identical excitation conditions (1 Hz square wave, 10 V RMS) are displayed in Figure 7. As Shore A hardness increases from 30A to 60A, the output signal amplitude exhibits a clear monotonic decrease, dropping by approximately 40–50% (from the peak-to-peak value observed at 30A to that at 60A). This trend arises because higher-hardness materials undergo significantly smaller axial compression δ under the fixed peak load F (Equation (14)), leading to a correspondingly smaller relative capacitance change ΔC/C_0_ (Equation (16)). The observed monotonic decrease is in excellent quantitative agreement with the force–capacitance coupling model (Equation (21)), confirming that the sensor can reliably transduce material stiffness variations into distinguishable electrical signals with high repeatability across multiple excitation cycles.

**Figure 8:**
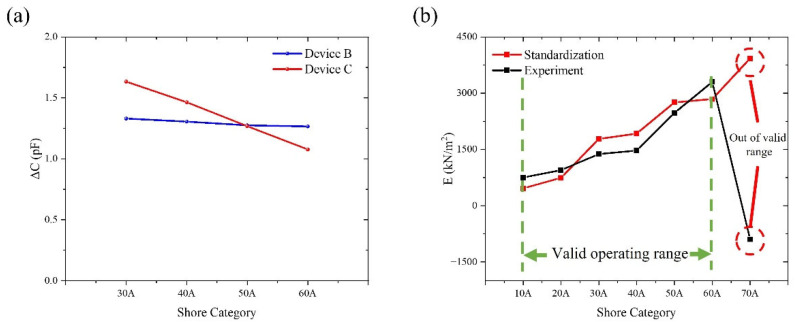
Repeatability test results are compared to system measurement and calibration data. (**a**) Extra ΔC comparison plots for devices A and B, displaying hardness block repeatability testing over the Shore 30A–Shore 60A range. (**b**) Comparison of experimental data with calibration data to observe their appropriate ranges.

To guarantee data comparability across several experiments, preprocessing, such as steady-state segment extraction and baseline correction, was applied to the obtained raw signals prior to data analysis. In order to reduce possible environmental effects on measurement results, external environmental variables were also quantitatively controlled during the experiments. These included laboratory temperature and humidity stability, vibration isolation of the test platform, and system shielding and grounding techniques. The system output signal maintained a full waveform shape and responded steadily to input fluctuations under applied square wave excitation. Under these excitation circumstances, the system showed reliable and consistent dynamic response capabilities. In order to preserve uniform installation techniques, contact circumstances, and loading parameters, repeat measurements were carried out for every standard block that represented a certain hardness grade. Seven hardness levels, ranging from Shore 10A to 70A, were included in this investigation. However, because of material deformation characteristics and contact boundary effects, the stability and repeatability of capacitive responses were reduced at the extreme low and high hardness values (Shore 10A, 20A, and 70A). This research focuses on the comparative examination of data from the Shore 30A–60A range, which shows superior signal quality, in order to guarantee the validity of system validation conclusions. It should be mentioned that the findings drawn from the system performance validation are unaffected by the unpresented extreme hardness grade data. Their main function is to describe the reaction boundaries in non-ideal contact situations; these findings will be investigated further in later research. The test findings for Device A and Device B, which were manufactured using the same procedures, show numerical discrepancies within the Shore 30A–60A range, but their variation patterns and response directions are still very consistent, as Figure 8a illustrates. When the standard block hardness grade varies, both Group A and Group B measurements show a roughly linear monotonic variation, with comparatively consistent rates of change between neighboring hardness grades. This suggests that the experimental system exhibits predictable and consistent response qualities to changes in the tested objects’ hardness. It should be highlighted that this study focuses on the relative trends and patterns of change in sensor output with hardness grade rather than attaining perfect numerical consistency between various experimental groups. There are a number of main reasons for the observed difference between Group A and Group B: First, repetitive manual clamping and unclamping necessarily generated some differences in contact states during the experiment, even with strict control over mounting techniques and loading circumstances. These include variances in the distribution of contact pressure and minute discrepancies in the sensor unit’s first contact position with the hardness block. Second, because the trials were carried out at several intervals, small variations in the surrounding temperature, humidity, and electromagnetic background could have a cumulative effect on the results of the measurements. Furthermore, during extended continuous operation, the sensing device itself displays some degree of sensitivity drift and zero-point drift, which is challenging to completely prevent in numerous repeated observations. The numerical magnitudes of Group A and Group B data reveal clear differences despite absolute value inconsistencies resulting from these factors, as illustrated in Figure 8a. Group A has a rather moderate changing trend, but Group B shows a more noticeable overall drop. However, there are no discernible aberrant swings or trend reversals, and both sets of data show a roughly linear monotonic relationship with changes in hardness block grade. It should be highlighted that the relative trend and rate of change features of sensor outputs as hardness fluctuates are the emphasis of this study, rather than the absolute consistency of measurement values between various experimental groups. Thus, Figure 8a successfully illustrates the experimental system’s linear response to hardness fluctuations and its exceptional repeatability, satisfying the validation requirements of this study even with amplitude discrepancies between Groups A and B. As a result, the experimental conclusion that the system is appropriate for characterizing hardness changes and trend identification is strongly supported by the data shown in Figure 8a.

Following system calibration using samples of different Shore hardness grades, the initial capacitance C_0_ at the initial overlap length L_0_ was determined using an RF analyzer. The relative change $\frac{\Delta C}{C_{0}}$ was then calculated from the acquired raw data. Based on the theoretical model established in Section 2, under small-strain approximation conditions, the inverse relationship between capacitance variation and axial loading force in the coaxial capacitive sensing structure can ultimately be confirmed and expressed as

(21) $$E=\frac{FL_{s}\alpha }{A_{s}\left(L_{0}\frac{\Delta C}{C_{0}}-H\right)}$$

Here, E represents the equivalent Young’s modulus of the tested sample, F denotes the applied external load, and α=4 is a correction parameter related to the device geometry and electromechanical coupling characteristics. This study performed parameter fitting and experimental validation of the above expression using experimental data within the 10A–70A range. The calibrated modulus of the standard sample and the inverted modulus results under various loading circumstances are compared in Figure 8b. The findings show: Normalized capacitance changes show a steady linear monotonic relationship with applied force within the 10A–60A range. For samples with different Shore A hardness, the inverted modulus findings exhibit consistent patterns with the calibration platform. The data points are concentrated in similar structural response regions, physically equivalent, and fairly distributed. However, these results only show the similarity and comparability of the response characteristics of the two systems and should not be viewed as strict model fitting, given the differences between the experimental and calibration platforms in structural assembly, electrode parameters, and testing environments. The sensing system functions within a stable linear strain response interval within this range, according to the inversion results for samples with different Shore A hardness, which are consistent with theoretical predictions. Although accurate quantitative characterization is not possible, this allows for trustworthy qualitative evaluation of the mechanical modulus of soft polymeric materials.

On the other hand, under Shore 70A circumstances, experimental data showed notable deviations: the relative capacitance change stopped following the established linear trend. The inverted Young’s modulus showed a significant discrepancy with calibrated values; the relative error increased noticeably and distorted the curve as a whole. The equivalent Young’s modulus inverted from capacitance changes produced non-physical negative values under Shore 70A settings. This event suggests that rather than being the result of data processing or computational errors, the experimental response varied from the applicable assumptions of the inversion model. According to the inversion formula $E=\frac{FL_{s}\alpha }{A\left(L_{0}\frac{\Delta C}{C_{0}}-H\right)}$, its physical validity relies on capacitance changes being primarily driven by axial compression of the sample and the system operating within the small-strain linear range. However, when applied to Shore 70A samples, the axial deformation of high-Shore-hardness specimens significantly diminishes and approaches the measurement resolution limit. Potential measurement deviations may cause $L_{0}\frac{\Delta C}{C_{0}}-H<0$, violations of the formula’s prerequisites result in a substantial offset. This makes it quite evident that the capacitive Young’s modulus measurement system’s effective testing range is between 10A and 60A. For later Young’s modulus inversion of unknown materials, this conclusion offers a solid basis. Additionally, it provides guidance on how to enhance electrode designs, close assembly gaps, and create large-strain corrective inversion models that can be used to shore materials with different levels of hardness [49].

Based on the fixed peak excitation force *F* calculated from the electromagnetic drive unit (Equation (14)) and the Young’s modulus inversion model (Equations (19) and (21)), the measurable range of Young’s modulus achievable by this system can be quantitatively defined as follows. Experimental calibration results show that in the Shore 10A–60A range, the inverted equivalent Young’s modulus stably distributes from 463 kPa (Shore 10A) to 2844 kPa (Shore 60A), which represents the actual measurable range of the current device configuration. To determine the physical limits of the system, combining the sensor resolution ($\delta_{min}$ ≈ $5\times 10^{-4}\times L_{0}$) and the upper limit of the small-strain linearity assumption ($\delta_{max}$ ≈ $0.025\;{\times L}_{0}\;$), the theoretical maximum Young’s modulus is $E_{max}$, and the theoretical minimum Young’s modulus is $E_{min}$. The above theoretical limits are fully consistent with the observed deviation at Shore 70A, further validating the physical applicability boundaries of this measurement system [5,50,51,52,53].

The experimental results demonstrate strong agreement with both theoretical predictions and independent calibration data. The monotonic decrease in signal amplitude with increasing Shore A hardness (Figure 7) directly reflects the inverse relationship between material stiffness and induced effective compression δ, as predicted by the electromechanical coupling model (Equation (21)). Quantitative comparison with the independent calibration platform (Table 1) shows that the measured Young’s modulus values are generally consistent with the Gent reference values (Gent, 1958 [48]; Equation (9)) across the 10A–60A range, with most data points lying within ±21% and an overall acceptable agreement for the majority of the hardness levels examined. At higher hardness (Shore 70A), however, the measured value deviates by –29%, consistent with the effective compression δ approaching the sensor resolution limit of 2.5 μm (Section 2.3). This systematic underestimation is attributable to the commercial elastomer blocks being slightly softer than the ideal natural rubber assumed in Gent’s model—a phenomenon also documented in dental elastomers (Meththananda et al., 2009 [16]). These findings align closely with recent studies on capacitive sensors for soft materials and electromagnetic actuation for elasticity testing, further validating the advantages of the present coaxial design in controllable transient loading and high-sensitivity detection. The observed deviations also underscore the system’s sensitivity to assembly precision, providing explicit guidance for future optimization.

It is worth noting that the proposed system does not replace but complements the standard Shore durometer (e.g., Teclock GS-719 or equivalent ASTM D2240-compliant instruments). The conventional Shore hardness value is obtained first with the commercial durometer, after which the same specimen is tested with the present electromagnetic–capacitive device to extract Young’s modulus. Calibration is performed only once using the commercial Shore A reference blocks (Table 2); for materials belonging to the same family, no recalibration is required for each new sample or batch. This feature significantly enhances practical applicability compared with methods that demand material-specific recalibration for every test [54].

## 4. Conclusions

In summary, this study presents a novel electromagnetic–capacitive coaxial system for the direct determination of Young’s modulus from Shore A hardness measurements. By integrating controllable Lorentz force actuation with high-sensitivity capacitive sensing in a compact modular architecture, the system achieves a displacement resolution of 2.5 μm and enables reliable modulus inversion over the Shore 10A–60A range without requiring large-scale instruments or destructive loading. The method is fully complementary to conventional Shore durometer testing (ASTM D2240): the standard Shore hardness value is first obtained with a commercial durometer, after which the same specimen is measured with the present system to extract the corresponding Young’s modulus. Calibration is performed only once using commercial Shore A reference blocks; subsequent measurements on materials of the same class do not require recalibration for each sample or each new batch, provided the material family remains consistent. Only when the material type changes substantially (e.g., from silicone rubber to polyurethane) is a single verification calibration recommended. This one-time calibration strategy, combined with the non-invasive and controllable nature of the excitation, makes the approach highly practical for both rapid laboratory characterization of engineering elastomers and potential non-invasive assessment of biological soft tissues. The results demonstrate good quantitative agreement with independent calibration data and Gent’s classical relation (Gent, 1958 [48]), confirming the system’s accuracy and reproducibility within its validated range. Future work will focus on further miniaturization and in situ application to real biological tissues, thereby extending the utility of hardness-based modulus evaluation beyond traditional laboratory settings [55].

## Figures and Tables

**Table 1:** Quantitative comparison of the present electromagnetic–capacitive system with representative existing methods for Young’s modulus measurement.

Measurement Method	Contact Mode	Typical Resolution	Typical Force Range	Equipment Cost ($)	Measurement Time per Sample	Suitability for Soft Tissue	Key Advantages of Present Method
Direct mechanical loading (tensile/compression)	Contact	~10 μm	0.1–100 N	5000–20,000	5–15 min	Low	Non-contact, controllable transient load, $\delta_{min}$ = 2.5 μm
Nanoindentation	Contact	1–10 nm	1 μN–10 mN	50,000–200,000	10–30 min	Medium	Macro-scale, no surface damage, $\delta_{min}$ = 2.5 μm
Friction/wear testing	Contact	~50 μm	0.5–50 N	10,000–30,000	10–20 min	Low	Direct E output, no friction model required
Ultrasound elastography	Probe contact	0.5–1 mm	N/A (acoustic)	50,000–150,000	1–5 min	High	Low-cost, portable, integrated
Magnetic resonance elastography	Non-contact	~1 mm	N/A (magnetic)	500,000–2000,000	15–60 min	High	Real-time, portable, no large imaging system
Impulse excitation technique (IET)	Mechanical impact	~20–100 μm	0.1–10 N	2000–10,000	1–3 min	Medium	Controllable electromagnetic actuation, repeatable
Present work: Electromagnetic–capacitive coaxial system	Electromagnetic non-contact + capacitive sensing	2.5 μm $\left(\delta_{min}\right)$	0.05–0.5 N	<2000	<30 s	High	Modular coaxial design, controllable transient loading, $\delta_{min}$ = 2.5 μm, suitable for non-invasive soft-tissue assessment

The present system resolution ($\delta_{min}$ = 2.5 μm) is calculated from the Wayne Kerr 6500B basic accuracy (±0.05%) and L_0_ = 5 mm (see Section 2.3). Cost estimates are approximate and based on commercial systems commonly used in research laboratories.

**Table 2:** Calibrated Young’s Modulus Values of Seven Shore Hardness Blocks in Comparison.

Shore Hardness(A)	Linear Strain Range (-)	Young’s Modules E (kPa)	Literature Reference Value E (kPa, Gent 1958 [48])	Relative Deviation (%)
10A	0–0.020	463	414	+12
20A	0–0.021	742	734	+1
30A	0–0.022	1780	1146	+55
40A	0–0.025	1923	1696	+13
50A	0–0.026	2756	2465	+12
60A	0–0.023	2844	3619	−21
70A	0–0.021	3929	5543	−29

Note: The Gent reference values were calculated using the above formula and have been converted to kPa; Relative deviation = (value from this study − reference value)/reference value × 100%.

## Data Availability

The original contributions presented in this study are included in the article. Further inquiries can be directed to the corresponding authors.
